# Recent Insights into Organoid-Derived Extracellular Vesicles and Their Biomedical Applications

**DOI:** 10.3390/jpm15100492

**Published:** 2025-10-14

**Authors:** Ahmed Abdal Dayem, Yeonjoo Kwak, Hyemin Jeun, Ssang-Goo Cho

**Affiliations:** Stem Cell and Regenerative Biotechnology Major, School of Advanced Biotechnology, College of Institute of Science and Technology, Molecular & Cellular Reprogramming Center, Institute of Advanced Regenerative Science, Institute of Health, Aging & Society, Konkuk University, Seoul 05029, Republic of Koreassangoo@konkuk.ac.kr (S.-G.C.)

**Keywords:** organoids, extracellular vesicles, three-dimensional, therapeutics, regeneration

## Abstract

Extracellular vesicles (EVs) play a crucial role in cell-to-cell communication by transporting functionally active molecules, including proteins, lipids, and nucleic acids. While extensive research has focused on EVs generated from traditional two-dimensional (2D) monolayer cultures (2D-EVs), the emergence of three-dimensional (3D) organoid systems has led to the development of organoid-derived EVs (OEVs), which more closely mimic the physiological conditions of native tissues. In contrast to 2D cultures, 3D systems offer improved EV yield and cargo specificity, enhancing their translational potential. This review discusses the distinctive features of OEVs, including their enhanced tissue relevance, diverse molecular composition, and promising therapeutic applications in areas like disease modeling, regenerative therapies, and targeted drug delivery. We also present an overview of the current organoid-based platforms used to produce OEVs, recent innovations in EV modification and bioengineering, and the practical barriers to their clinical adoption. By comparing the strengths and limitations of OEVs with those of 2D-EVs, we provide a comprehensive perspective on their future role in precision healthcare, biomarker identification, and advanced therapeutic strategies.

## 1. Introduction

Traditional cell lines have been central to biomedical research and have contributed to numerous medical advancements in recent decades. However, their limited capacity for differentiation and single-lineage nature restrict their use in complex drug testing and studies on human organ development [1]. The emergence and ongoing refinement of organoid technology, supported by stem cell advancements, offers a promising alternative for addressing these challenges in modern biomedical research.

In recent years, significant progress has been made in controlling cellular behavior at the molecular level, enabling stem cells to self-organize and differentiate into specific cell types for organ repair [2,3]. Two-dimensional (2D) cell culture systems have served as a cornerstone for fundamental research and therapeutic exploration [4]. However, their limitations have become increasingly apparent.

Traditional 2D cultures lack essential features of native tissues, including the extracellular matrix (ECM), biochemical signals, and dynamic environmental cues, which are vital for replicating natural cellular behavior [5]. As a result, there is a growing demand for more sophisticated platforms for in vitro studies. The development of three-dimensional (3D) culture models has advanced, offering improved biological relevance by better preserving tissue-specific functions. Among these, organoid systems have emerged as particularly promising, as they more closely resemble the physiological characteristics of real tissues and organs compared to conventional models [6].

3D organoid cultures provide a physiologically relevant microenvironment that enhances cell–cell communication and functional maturation, thereby yielding extracellular vesicles (EVs) with distinct and superior properties compared to those derived from traditional 2D systems [7,8]. Notably, several structures described in previous reports technically meet the criteria for spheroids, as they overlap in biological characteristics with organoids. However, not all spheroids meet the criteria for classification as organoids [4]. Some lack an extracellular scaffold, do not exhibit sustained in vitro expansion, or consist of heterogeneous and disorganized cell populations. The key distinction lies in organoids’ ability to mimic the structural and functional characteristics of native tissues.

In contrast, spheroids are generally simpler, exhibit limited functionality, and are maintained for shorter culture durations [9,10]. While spheroids are generally used for short-term studies and not maintained for extended periods, organoids are often cultured long-term and can be cryopreserved for future applications [1,11,12].

On the other hand, assembloids are 3D structures composed of multiple cell types embedded within an extracellular scaffold, commonly used to study tissue-specific cellular interactions [13,14]. Despite sometimes being mistaken for spheroids due to their shape, assembloids are structurally more complex and a better mimic of in vivo tissues [15].

**Figure 1:**
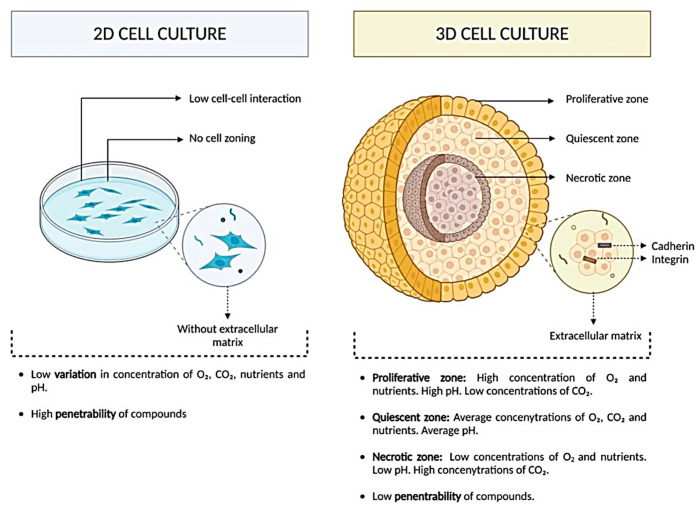
Comparative overview of 2D versus 3D cell culture systems. In 2D cultures, cells grow as flat monolayers without extracellular matrix support, exhibiting limited cell–cell interaction, no zonal organization, low variation in nutrient/gas gradients, and high compound penetrability. In 3D cultures establish proliferative, quiescent, and necrotic zones, with cells embedded in an extracellular matrix and connected through cadherins and integrins. These systems generate physiologically relevant gradients of oxygen, CO_2_, nutrients, and pH, while reducing compound penetrability, thereby more closely mimicking native tissue microenvironments. This figure was reproduced from ref. [16]. © 2024 by the authors. Licensee MDPI, Basel, Switzerland. This article is an open access article distributed under the terms and conditions of the Creative Commons Attribution (CC BY) license (https://creativecommons.org/licenses/by/4.0/).

Traditional 2D cultures, while widely used, fail to capture the structural and biochemical complexity of native tissues, limiting their ability to mimic in vivo physiology (Figure 1). In contrast, 3D culture-based systems recreate a more physiologically relevant microenvironment by enabling spatial cell organization, extracellular matrix (ECM) interactions, and the formation of proliferative, quiescent, and necrotic zones (Figure 1). These features support gradients of oxygen, nutrients, and metabolites that better reflect tissue architecture, thereby enhancing the predictive value of 3D models for regenerative medicine, drug testing, and disease modeling (Figure 1).

Mesenchymal stem cells (MSCs), derived from sources such as adipose tissue, umbilical cord, bone marrow, and dental pulp, are known for their therapeutic applications [17,18,19]. Conventional 2D monolayer cultures, though widely used for expanding MSCs, fail to replicate the native in vivo microenvironment. This can impair cell proliferation, reduce multipotency, induce chromosomal instability, and accelerate senescence and differentiation. In contrast, 3D culture systems more accurately mimic physiological conditions [20]. ECM-based hydrogels and aligned nanofibers used in 3D cultures enhance cytokine and growth factor secretion, promote angiogenesis, and reduce inflammation. As a result, MSCs cultured in 3D environments exhibit enhanced osteogenic and angiogenic activity, more closely mimicking in vivo conditions [21].

Advances in bioengineering and biomaterials have driven the development of organoid technology, providing more physiologically relevant models than traditional 2D cultures [5,22,23,24]. Organoids replicate the structure and function of native tissues, possess stable, self-renewing stem cells, and can be precisely manipulated at multiple biological levels [25]. In contrast to stem cell therapies, which often face challenges such as immune rejection, tumorigenicity, and functional limitations, organoids can more effectively mimic human organ architecture and function [26]. As such, organoid platforms have shown great potential in studying development, disease mechanisms, drug screening, and reducing reliance on animal models in biomedical research [27,28,29,30,31,32].

Organoids are sophisticated 3D culture systems sourced from either induced pluripotent stem cells (iPSCs) or tissue (adult stem cells). Organoids generated from healthy or diseased donor tissue preserve the biological traits of the original tissue, making them particularly useful for investigating tissue development and modeling various diseases [5,22]. As such, they span the gap between 2D cell cultures and intricate animal models, emerging as a robust platform for disease research and pharmaceutical development [33].

EVs are nanovesicles classically measuring 20–150 nm in diameter, emanating from the endosomal membrane system and secreted through exocytosis [34]. EVs were first discovered in the early 1980s in reticulocytes as vesicles containing the transferrin receptor [35,36,37]. EVs facilitate intercellular communication via paracrine and endocrine mechanisms [34,38] and modulate both physiological activities and pathological conditions [39]. Notably, EVs transport a wide range of biomolecules, including mRNA, miRNA, proteins, and lipids, positioning them as promising candidates for both therapeutic and diagnostic applications [40]. Their potential applications range from serving as nanocarriers for targeted drug delivery to acting as biomarkers and therapeutic agents [41,42,43].

**Figure 2:**
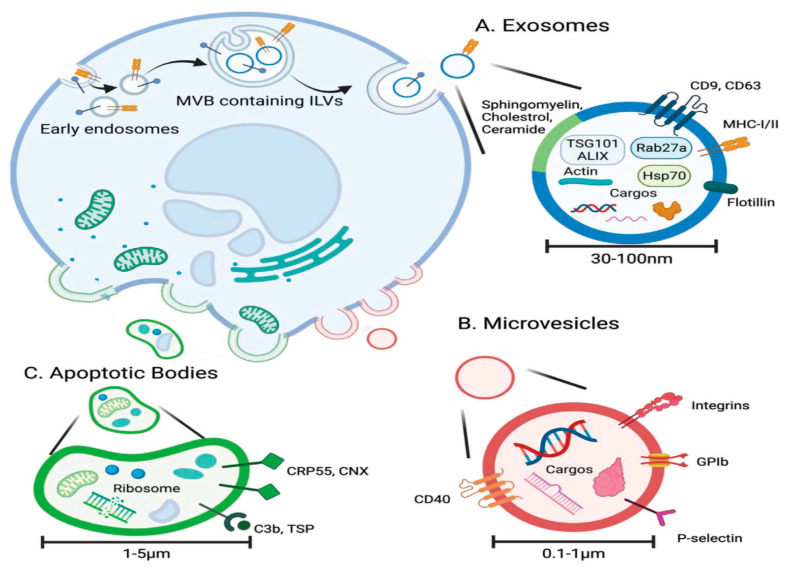
The biogenesis and characteristics of extracellular vesicles. (**A**) Exosomes originate from intraluminal vesicles (ILVs) within multivesicular bodies and are secreted with a cargo of proteins, lipids, and nucleic acids. (**B**) Microvesicles bud directly from the plasma membrane, carrying similar biomolecules. (**C**) Apoptotic bodies form during programmed cell death, also by membrane budding, and typically contain fragmented organelles and cellular debris. This figure is reproduced from ref. [52] with permission. © 2022 The Authors. Advanced Science published by Wiley-VCH GmbH. This is an open access article distributed under the terms of the Creative Commons CC BY license, which permits unrestricted use, distribution, and reproduction in any medium, provided the original work is properly cited. http://creativecommons.org/licenses/by/4.0/.

Exosomes are released through the fusion of endosomes with the plasma membrane [36], whereas microvesicles and apoptotic bodies are formed by outward budding directly from the plasma membrane [44]. Early endosomes are formed by the inward budding of the plasma membrane at the initial stage of exosome biogenesis [45], which mature into multivesicular bodies (MVBs) (Figure 2). These MVBs generate intraluminal vesicles (ILVs) enriched with lipids, proteins, and nucleic acids [46]. ILV formation is largely mediated by the endosomal sorting complex required for transport (ESCRT) complex and its associated proteins, including tumor susceptibility gene 101 (TSG101) and associated proteins ALG2-interacting protein X (ALIX), which cluster ubiquitylated proteins and facilitate inward budding of the endosomal membrane [46].

Ceramide lipids build lipid raft microdomains, which trigger MVB membrane budding, representing a distinct ILV formation mechanism [47]. Exosome release occurs constitutively through the trans-Golgi network or in a controlled manner through MVB–plasma membrane binding [45]. Rab proteins (Rab27a/b) facilitate the binding of MVBs to the plasma membrane, and SNARE proteins mediate the fusion of the endosomal and plasma membranes [48]. Microvesicle release, on the other hand, involves budding from the plasma membrane without exocytosis and is less sophisticated than exosome secretion [49]. It has been proposed that ESCRT-dependent viral-like budding or budding driven by a cytoskeletal imbalance caused by lipid translocation plays a role in microvesicle budding and breaking off [50]. Apoptotic bodies, formed during late-stage apoptosis via membrane blebbing, enclose fragmented cellular components and are rich in organelles, aiding their isolation [44,51].

The International Society for Extracellular Vesicles (ISEV) has established the minimal experimental criteria necessary for the identification and functional analysis of EVs, while also stressing the importance of standardization in EV characterization procedures [38,53,54]. A recent report highlighted both the technical challenges associated with EV isolation and the pressing need for consistent methodologies across studies [55]. Moreover, a thorough understanding of EV biology, including their roles in intercellular communication, physiological regulation, and disease mechanisms, underscores the critical impact of the chosen isolation techniques [34,55].

Characterization of EVs is carried out through the measurement of the expression level of various specific surface and cytosolic protein markers, such as Alix, Hsp70, CD9, CD63, CD81, TSG101, and MHC molecules, as well as by morphology using electron microscopy techniques (TEM, SEM, cryo-EM) and atomic force microscopy [56]. EV size is typically analyzed using nanoparticle tracking analysis (NTA) or dynamic light scattering (DLS). Additionally, fluorescent dyes such as PKH26, PKH67, 1,1′-dioctadecyl-3,3,3′,3′-tetramethylindo-carbocyanine perchlorate (DIL), 2-(5-(1,3-dihydro-3,3-dimethyl-1-octadecyl-2H-indol-2-ylidene)-1,3-pentadienyl)-3,3-dimethyl-1-octadecyl-perchlorate (DID), and Mem Dyes are employed to label exosomes for cellular uptake and tracking studies [57]. Additionally, the ISEV has recommended that the generic term “EVs” should be used unless the vesicle’s specific biogenesis pathway has conclusively identified [54]. For example, exosomes originate from multivesicular endosomes, whereas microvesicles are released directly from the plasma membrane. EVs may also be classified by physical properties such as particle size or the method of isolation, using descriptors like “small EVs” (under 200 nm) and “large EVs” (over 200 nm). This addresses the inconsistencies in EV terminology throughout the scientific literature [58].

3D cell culture systems have advanced the study of EVs, revealing unique molecular signatures and enhanced therapeutic properties, such as anti-inflammatory and pro-angiogenic effects [59,60,61]. Unlike EVs from 2D cultures, organoid-derived EVs (OEVs) exhibit superior biological complexity and functionality, making them promising candidates for clinical applications.

Based on accumulating evidence, we hypothesize that OEVs represent promising therapeutic candidates, though their translation into clinical practice requires rigorous validation. OEVs are emerging as promising platforms that recapitulate complex tissue environments and serve as therapeutic cargo for regenerative medicine [62,63]. By delivering proteins, nucleic acids, and lipids, they can enhance the maturation of organoids and therapeutic efficacy [64]. Notably, OEVs often exhibit more substantial physiological effects and higher yields than conventional EVs, making them attractive for applications such as drug delivery, regenerative therapy, and precision medicine [65]. However, key challenges remain, including limited production yields, a lack of standardized isolation and characterization protocols, restricted targeting specificity, and an incomplete understanding of the function of EV cargo [63,66,67]. Scaling up OEV production, ensuring reproducibility, and navigating regulatory hurdles are additional barriers. Overcoming these gaps will require deeper mechanistic studies, advances in biomanufacturing and cargo engineering, and robust translational research to enable clinical adoption.

Recent advances in organoid platforms have revealed their capacity to secrete EVs that recapitulate many of the structural and functional attributes of native tissue-derived EVs. Given their enhanced physiological relevance, molecular complexity, and tissue specificity compared to conventional 2D culture-derived EVs, we hypothesize that OEVs represent a promising next-generation platform for regenerative medicine, targeted drug delivery, and disease modeling. This hypothesis serves as a foundation for the subsequent discussion of therapeutic applications, where we critically evaluate emerging preclinical and translational evidence supporting the potential of OEVs in precision medicine.

This review compares EVs from 2D and 3D cultures, highlights the distinct advantages of OEVs, and discusses their emerging roles in biomarker discovery, precision medicine, and future therapeutic strategies.

## 2. A Synopsis of Organoids

### 2.1. Sources

Organoids are 3D cellular aggregates that develop and differentiate within an in vitro environment, mirroring the structural organization and physiological functions of real organs [68,69,70]. Despite significant progress, organoid research remains in an early stage [65]. The long-term aim of organoid technology is to engineer systems with multiple integrated functions, such as bone formation, bone resorption, hematopoiesis, and mechanical strength, to more effectively replicate the complexity of native tissues and improve clinical outcomes [71].

Organoids can be generated from various sources, including embryonic stem cells (ESCs), iPSCs, and adult stem cells (ASCs) [72,73]. Their formation relies on spatially regulated lineage specification and cellular self-sorting, guided by tailored culture conditions and signaling factors [74].

ASCs, which are derived from adult tissues such as skin or blood, possess limited differentiation capacity, typically giving rise only to cell types that are native to their tissue of origin [75,76]. They play a crucial role in maintaining tissue integrity, promoting growth, and facilitating regeneration. Notably, ASCs present minor ethical concerns, as they are sourced from adult tissues rather than embryonic origins [25]. However, their limited differentiation capacity restricts ASC-derived organoids to primarily a single epithelial lineage [5]. On the other hand, ASC-derived organoids afford a more straightforward and time-efficient approach, with maturity levels comparable to those of native adult tissues, making them feasible for tissue repair applications [2]. Moreover, they can be expanded in vitro over extended periods while maintaining genomic stability [2]. ASC-organoids replicate both the structure and function of native tissues via utilizing stem cell-based technologies [77].

Directed stimulation of ASCs with various growth factors or small molecules resulted in their multiplication and differentiation into specialized cells, contributing significantly to tissue repair and renewal following damage or disease [78]. As a result, ASC-derived organoids tend to reflect limited organ-specific functionality. Nevertheless, ASCs are favored for their accessibility and abundance, making them a practical choice for organoid generation. In contrast, PSCs exhibit a broad differentiation capacity, enabling them to generate a wide variety of cell types that resemble those found throughout the human body [79,80,81].

Additionally, iPSC-derived organoids exhibit a complex spatial organization and encompass a broader spectrum of physiological functions, thereby enhancing their overall functional diversity [82,83]. Therefore, PSC-derived organoids are particularly well-suited for studying early organ development [5], as well as for applications in basic research and drug discovery [84,85]. iPSCs offer distinct ethical benefits, as they can be generated from any adult somatic cell type, bypassing the need for embryonic sources. Despite their promise, iPSC-derived organoids often exhibit limited capacity for sustained growth and carry a risk of teratoma formation upon transplantation [25]. For instance, a research team optimized the culture conditions for human iPSC-derived midbrain organoids over various time points [86]. These organoids were later integrated into striatal neural circuits and used to alleviate symptoms of Parkinson’s disease in a mouse model. Additionally, the study addressed the tumorigenic risks associated with iPSCs, offering strategies to mitigate such concerns [86]. This highlights the potential of iPSCs to generate organ structures.

In summary, organoids derived from ASCs and PSCs each present unique advantages for tissue regeneration and functional restoration. However, their respective strengths and limitations should be considered for the specific application. Importantly, the cellular origin of organoids can significantly affect both the quality and yield of the EVs they produce, which in turn influences their therapeutic potential, a topic explored in the following sections.

### 2.2. Culture Methods

#### 2.2.1. Bioreactors

Bioreactor systems create a regulated 3D environment that enhances cell proliferation, development, and self-organization within organoids, resulting in more physiologically relevant models [87,88].

Rotating bioreactors, liquid-filled cylinders rotating on a shaft, promote efficient material diffusion and support the growth of nonvascular 3D organoids [87]. This method is relatively simple and enhances both the structural integrity and morphology of organoids [89]. For example, one study demonstrated the scalable production of kidney organoids using a low-cost 125 mL rotating bioreactor [90], while another research team generated forebrain-specific organoids mimicking cerebral cortex development [91]. A recent report by Cohen et al. utilized a stirred-tank bioreactor to achieve a 277-fold expansion of hPSCs in just 6.5 days [92].

The bioreactor-based platforms have demonstrated the promising potential of bioreactors for large-scale, uniform, and efficient cultivation of organoids. Table 1 summarizes the characteristics of commonly used bioreactor platforms, providing a framework for selecting suitable systems for specific experimental and translational needs.

#### 2.2.2. Microfluidics

Microfluidic technology offers a reliable platform for organoid formation and maturation, facilitating intercellular communication and providing a consistent microenvironment with essential chemical and mechanical cues [106]. Microfluidic platform offers more scalable organoid production compared to conventional methods [107]. Additionally, when integrated with “organoids-on-a-chip,” it supports multi-organ modeling on a single device [108]. For example, one research team developed a chip containing over seven retinal cell types [103], while another demonstrated engineered multilayer microfluidic systems for generating and real-time monitoring islet organoids [109].

The incorporation of biomimetic materials into microfluidic platforms has significantly expanded their biomedical applications beyond traditional capabilities. Although traditional static and scaffold-based systems have been foundational for therapeutic studies, they remain limited by several critical constraints. These include the inability to precisely control growth factor gradients, oxygen/nutrient diffusion, and mechanical stimulation, often leading to heterogeneous differentiation, necrotic cores in spheroids/organoids, and poor reproducibility across experiments [110,111,112]. Moreover, conventional pellet and 2D cultures provide limited opportunities for dynamic monitoring, making them unsuitable for high-throughput screening or real-time assessment of cell behavior [113,114,115].

Microfluidic platforms effectively address these limitations by integrating fine spatiotemporal control over both biochemical and biophysical cues. These systems enable the establishment of stable concentration gradients, dynamic perfusion mimicking vascular supply, and controlled shear forces that more accurately reproduce the native microenvironment [116,117,118]. Furthermore, the miniaturized scale of microfluidic devices allows real-time imaging and high-throughput testing, facilitating both fundamental studies of stem cell chondrogenesis and translational applications in drug discovery and regenerative medicine [119,120,121].

For instance, a research team developed a collagen-based bone-on-a-chip using pre-differentiated adipose-derived stem cells, marking the first miniaturized 3D bone model for personalized tissue engineering [122]. While microfluidics and hydrogels have advanced organoid-based tissue repair models by streamlining processes and enhancing culture efficiency, large-scale production remains a challenge. To address this, a study introduced a scalable microfluidic system for generating vascularized hepatobiliary organoids from hPSCs [123].

Recent advancements in brain organoid research have utilized microfluidic systems combined with decellularized brain ECM to establish 3D microenvironments, supporting in vitro maturation of iPSC-derived organoids for neuroscience and disease modeling [124]. Similar approaches using hydrogels and microfluidics have enhanced intestinal organoid development by promoting stem cell organization and epithelial formation [125]. Additionally, microfluidic platforms have enabled the creation of retinal organoids comprising multiple cell types and improved islet organoid viability, thereby expanding the applications in retinal and diabetes research. Overall, microfluidics is revolutionizing organoid research by enabling the development of multi-organ systems for regenerative and therapeutic studies.

#### 2.2.3. Microwell

Microwell-based techniques, including photolithography and micropatterning, facilitate the formation of 3D multicellular structures by enabling precise cell aggregation within microscale wells. This method enables accurate, scalable, and cost-effective organoid formation [2,108,126]. In addition, it utilizes microscale porous architectures to regulate cell positioning and development in 3D space, thereby creating an optimal physical microenvironment for the formation of organoids [127].

The design of microporous array design, including pore size, spacing, and surface chemistry, can be tailored to control cell adhesion and distribution. These structures support 3D cellular growth and enhance tissue-specific traits by fostering cell–cell interactions. Notably, they have been shown to aid in the development of polarity, lumen formation, and neural network organization in intestinal, neural, and liver organoid models [70,128].

One research team utilized microwell arrays to generate brain organoids with optimized shape, size, and structural features, including sulcus formation and defined neuronal layers [128]. Microwell arrays enhance the uniformity of organoid-based disease models. For example, a team applied low-adhesion 96-well plates to initiate human midbrain organoids, later transferring them to ultra-low-adhesion six-well plates for final differentiation [86]. Similarly, another study demonstrated the utilization of porous chitosan microspheres with evenly distributed micropores to generate consistent spinal cord organoids [129].

Despite their advantages, microwell systems involve labor-intensive procedures, highlighting the potential value of integrating AI and robotics for more efficient organoid culture.

#### 2.2.4. Hydrogel

Utilizing hydrogel-based matrices in organoid culture offers robust structural support for stem cell self-assembly. Due to their excellent biocompatibility and adjustable physical and chemical characteristics, hydrogels effectively emulate the ECM, creating a more physiologically relevant environment for organoid development [130,131,132]. The emergence of stem cell–matrix-based hydrogel enhances the physiological relevance of organoids [130]. As our understanding of the ECM grows, customized hydrogels with 3D structures and adaptable features are increasingly favored for organoid culture due to their ability to more effectively replicate in vivo conditions [133,134]. Integrating hydrogels with natural (e.g., gelatin, fibrin) or synthetic (e.g., polylactic acid) materials enhances their mechanical strength and cell adhesion while preserving biodegradability. This enhances the structural stability of organoids and promotes their growth and differentiation [135,136].

For example, a group of scientists developed bone organoids using temperature-responsive poly(*N*-isopropylacrylamide) (pNIPAAm) hydrogels to generate spheroids from BMSCs and BMSCs/DPSCs [137]. Similarly, another research team used hESCs and iPSCs in four-armed maleimide-capped polyethylene glycol (PEG-4MAL) hydrogels to generate intestinal organoids, which enhanced colonic wound healing when delivered via colonoscopy [138]. This synthetic hydrogel is a promising alternative to Matrigel for organoid culture. A study demonstrated that peptide-based hydrogels with controlled stiffness support the maturation of hiPSC-derived kidney organoids [139]. Furthermore, a research team showed that hyaluronic acid hydrogels with liver-like viscoelasticity promote cholangiocyte organoid growth and upregulate yes-associated protein (YAP) expression [140]. These studies underscore the significance of engineered microenvironments in influencing organoid development and function.

#### 2.2.5. Bioprinting

3D bioprinting has emerged as a robust tool for organoid fabrication, utilizing bioinks that contain cells and extracellular matrix components, which solidify into stable 3D structures under controlled conditions [141]. This technique enables the precise and reproducible construction of organoids with native-like architecture and function, advancing applications in regenerative medicine, disease modeling, and personalized therapy.

**Figure 3:**
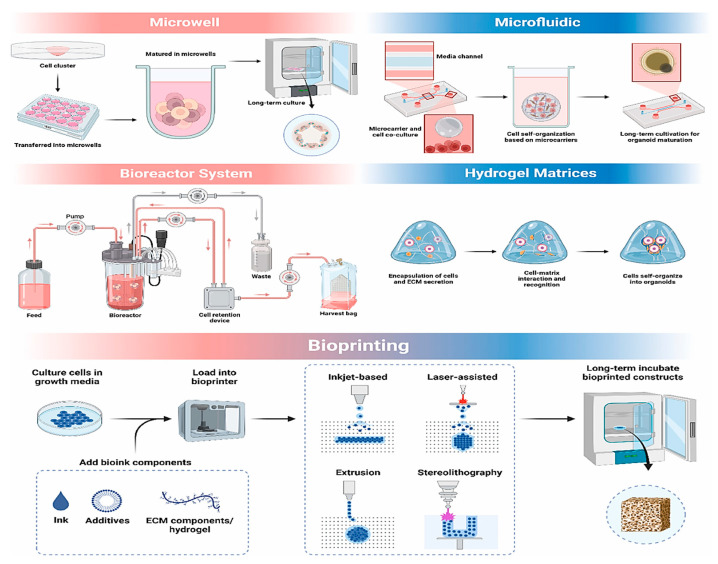
Schematic diagram illustrating common 3D culture strategies, including microwell arrays, microfluidic platforms, bioreactors, hydrogel scaffolds, and bioprinting techniques, can mimic the in vivo microenvironment, offering optimal conditions that promote organoid development, functional maturation, and overall structural complexity. This figure is reproduced from ref. [7] with permission. © 2025 The Authors. Published by Elsevier Ltd.

Bioprinting enables precise spatial control of cells, facilitating the creation of multilayered, functional organoids, as shown in studies where a research team developed self-mineralizing bone organoids [142], and Yang et al. printed liver organoids with physiological functions [143]. Despite its advantages, challenges remain, particularly regarding cell viability during printing and achieving effective vascularization. Notably, 4D printing, a promising advancement, enables biomaterials to morph dynamically in response to stimuli, offering enhanced physiological relevance and improved organoid stability [144]. Figure 3 outlines the main approaches for 3D culture.

### 2.3. Therapeutic Applications

Organoids, as 3D cell clusters composed of diverse cell types and layered architectures, afford a more physiologically relevant in vitro model than traditional repair materials by closely replicating native tissue structure and function. They recreate organ-specific anatomy and partially restore physiological activities, making them uniquely suited for tissue repair applications. Organoids coordinate multiple cell types, which are especially beneficial for repairing multicellular tissues, such as skin, liver, and intestines. Their versatility enables them to play roles in cell replacement, functional recovery, and structural repair. Furthermore, when transplanted into injury sites, organoids not only offer scaffolding but also promote tissue integration and communication with host cells, enhancing repair outcomes.

Inflammatory bowel disease (IBD), including Crohn’s disease and ulcerative colitis, is becoming a global health concern. Although the prevalence in Western countries now exceeds 0.3%, newly industrialized regions in Asia and South America are seeing a sharp rise in incidence [145,146]. The chronic, relapsing course of IBD, along with complications such as intestinal fibrosis and an increased risk of colorectal cancer, poses significant clinical challenges [147,148]. Conventional research approaches, including animal models and 2D cell cultures, often lack translational accuracy. This is primarily due to species-specific physiological differences and their inability to faithfully mimic the structural and immunological complexity of the human gut [149]. For example, animal models cannot fully reproduce the genetic heterogeneity and microbial ecology observed in human IBD, while 2D epithelial monolayers fall short in recapitulating the multicellular organization of the intestinal microenvironment [150]. These limitations underscore the urgent need for more sophisticated model systems that can effectively bridge the gap between preclinical research and clinical application.

Intestinal organoids-organizing 3D structures derived from adult or pluripotent stem cells have significantly advanced IBD research [151]. For instance, a research team developed colon organoids derived from leucine-rich repeat-containing G protein-coupled receptor 5 (Lgr5)-positive intestinal stem cells [152]. These organoids were dissociated and delivered via enema to inflamed intestinal regions in an IBD mouse model, where they successfully engrafted and contributed to partial symptom relief. Post-transplantation analysis revealed increased expression of Ki67, Alcian blue, chromogranin A (ChgA), carbonic anhydrase II (CA2), and cytochrome c oxidase subunit I (COX1), indicating enhanced epithelial regeneration, mucus production, and improved intestinal metabolism in affected tissues [152].

In 2022, the world’s first clinical use of patient-derived intestinal organoids to treat IBD was reported [153]. Notably, no additional drug therapy was administered, and the patient demonstrated initial signs of recovery, though long-term outcomes remain under observation. According to the Japan Clinical Trial Registry (ID: jRCT b032190207), the university has initiated a clinical trial to evaluate the safety of autologous intestinal epithelial stem cell transplantation in patients with refractory ulcerative colitis [153]. The trial will involve eight participants aged 20 or older. Autologous epithelial organoids will be cultured ex vivo for approximately five weeks and, upon confirming viability, will be endoscopically transplanted into ulcerated colonic areas.

Traumatic brain injury often results in persistent neurological deficits [154,155]. In the adult mammalian brain, both neurogenesis and axonal regeneration are limited and occur in restricted regions [156,157]. Studies have shown that cell transplantation offers promise for neural restoration. For instance, grafting fetal rodent cortical tissue into the adult cortex has been found to promote neuronal outgrowth and establish localized integration within the host brain. These findings underscore the potential of cellular transplantation approaches to re-establish neural circuits in the cerebral cortex.

Brain organoids, a type of 3D in vitro neural models, provide a powerful platform for investigating brain repair and neuro-regeneration due to their capacity to closely mimic brain development, cellular diversity, and functionality. In traumatic brain injury, they can regenerate damaged neurons and glial cells while promoting endogenous repair through the action of neurotrophic factors [158]. For stroke-related neuronal loss, vascularized brain organoids serve both as viable donor tissue and as tools for reconstructing damaged brain regions [159]. Additionally, in neurodegenerative diseases, they hold potential for replacing lost dopamine neurons and restoring neural circuits [86].

A research team developed an efficient protocol that enhanced brain organoid development by activating Wnt and ERK signaling while suppressing SMAD and TGF-β pathways [158]. After 80 days of culture and transplantation into the adult visual cortex, the organoids achieved an 82.1% graft survival rate and partially restored visual signal processing. Moreover, another research group utilized low-intensity ultrasound (LIUS) to accelerate cortical organoid maturation and integration into the somatosensory cortex [160]. Furthermore, organic brain–computer interfaces (OBCIs) have emerged as tools to promote organoid development and closed-loop functional integration with host brain circuits, offering promising strategies for targeted neural repair [161].

Collectively, organoid-based models represent a promising complement to conventional tissue repair strategies, owing to their cellular precision, structural complexity, and ability to restore tissue function. While this review does not aim to comprehensively cover all the therapeutic and regenerative applications of organoids, it is worth noting that they hold considerable potential, particularly in addressing challenging scenarios for tissue repair. For readers seeking a broader overview, several excellent review articles are available that thoroughly discuss the therapeutic applications of organoids [7,162,163,164].

## 3. Extracellular Vesicles from 2D and 3D Cell Culture Systems

**Figure 4:**
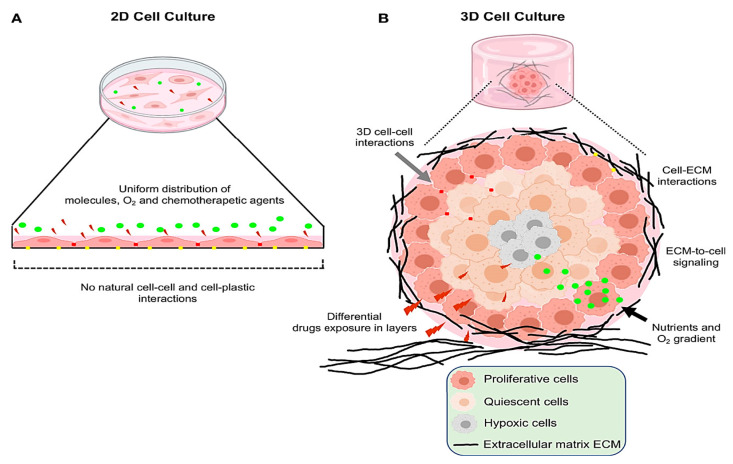
Representative figure depicting the key differences between the 2D and 3D cell culture models. (**A**) In conventional 2D cultures, cells form a flat monolayer on plastic surfaces, with minimal cell–cell interaction and unrestricted access to nutrients, oxygen, and drug conditions that poorly mimic in vivo environments. (**B**) 3D cultures foster enhanced interactions among cells and with the extracellular matrix, while limiting nutrient and oxygen diffusion. This structural complexity more accurately reflects the tumor microenvironment seen in living organisms. This figure is reproduced from ref. [172]. Copyright © 2022 Salinas-Vera, Valdés, Pérez-Navarro, Mandujano-Lazaro, Marchat, Ramos-Payán, Nuñez-Olvera, Pérez-Plascencia and López-Camarillo. This is an open-access article distributed under the terms of the Creative Commons Attribution License (CC BY).

Cell cultured in 2D systems undergo morphological changes and cytoskeletal reorganizations, acquiring artificial polarization that ultimately results in abnormal gene and protein expression [165,166]. Conversely, 3D conditions enhance interactions between cells and the extracellular matrix (Figure 4) [167]. The characteristics of the in vivo environment, including cell heterogeneity, hypoxia, growth dynamics, signaling system activity, and gene expression patterns, are more accurately reflected by this culture model [168,169]. Furthermore, 3D cultures provide a perfect model to examine the behavior of tumor cells because they preserve the shape and polarity of the cells while generating a gradient of O_2_, nutrients, and metabolic waste (Figure 4) [170,171].

A consistent and scalable source of MSCs, the main source of EVs, is crucial to fully harnessing their therapeutic benefits. However, conventional 2D culture systems used for EV production face significant limitations, including low yield and reduced cell functionality due to the senescence of MSC. This senescence impairs regenerative properties, including proliferation, differentiation, migration, and angiogenic potential [173,174,175].

The absence of cell polarity and impaired extracellular interactions, in a 2D culture platform, disrupt cellular architecture and alter responses to cellular events, such as apoptosis [176,177,178]. These conditions fail to replicate the native MSC niche, limiting the therapeutic value of the produced EVs. To address these limitations, advanced preconditioning methods, such as 3D culture and hypoxia, have been introduced [61]. Among them, 3D culture better maintains MSC stemness, enhances EV yield, and improves the functional quality of EV cargo, thereby increasing overall therapeutic potential [179,180].

Proteins and miRNAs that are crucial to energy metabolism, immunomodulation, bone development, cell division, and neuronal activity are plentiful in 3D-EVs [60,181]. Additionally, 3D-EVs exhibit superior anti-inflammatory and anti-apoptotic activities, indicating stronger therapeutic potential [182]. Notably, 3D cultures using hollow fiber bioreactors significantly increase EV yield from umbilical cord-derived MSCs (UC-MSCs) [180], producing vesicles with enhanced cardioprotective and renal protective effects, including mitigation of cisplatin-induced acute kidney injury [61].

Both scaffold-free and scaffold-based 3D cell culture systems more closely mimic in vivo environments, resulting in enhanced EV production and bioactivity [183]. For instance, 3D-EVs from hUMSCs cultured on a graphene scaffold more effectively reduced Aβ accumulation and improved cognitive function in APP/PS1 mice, linked to elevated expression of Aβ-clearing enzymes and reduced inflammation and oxidative stress [184]. In contrast to 2D-EVs, 3D-EVs exbibit unique protein profiles and molecular signaling [20,185] and demonstrate greater therapeutic properties, including improved anti-inflammatory, pro-angiogenic, and regenerative activities [59,60,61].

Moreover, 3D culture-based methods enable more efficient cell expansion and cost-effective scalability, resulting in significantly higher EV yields, a crucial factor for clinical translation. For example, hMSCs cultured in a 3D dynamic wave-motion bioreactor produced approximately twice as many EVs as in 2D settings, along with smaller vesicles and increased expression of EV biogenesis genes from both ESCRT-dependent and -independent pathways [186]. Similarly, human bone marrow-derived MSCs (hBM-MSCs) cultured in 3D vertical-wheel bioreactors with microcarriers yielded 24 times more EVs and demonstrated a greater capacity to stimulate neurite extension [187].

3D culture-derived EVs (3D-EVs) differ significantly from 2D-EVs in terms of molecular signaling and protein composition [17,152], and have demonstrated superior therapeutic potential, including enhanced angiogenic, regenerative, and anti-inflammatory effects [59,60,61]. Three-dimensional systems also enable more efficient cell expansion, improving EV yield and scalability for clinical applications [188]. For instance, human MSCs cultured in 3D wave-motion bioreactors produced approximately twice as many, smaller EVs, with upregulated genes involved in both ESCRT-dependent and -independent biogenesis pathways [186]. Another research team has demonstrated up to 24-fold increases in EV yield using 3D bioreactors or scaffolds, along with greater efficacy in promoting neurite outgrowth, wound healing, and neuroregeneration compared to EVs from 2D cultures [187].

3D-cultured hMSCs demonstrated a significant improvement in EV production and increased CD63 expression [186]. Another study reported that 3D-MSCs release EVs with 28-fold higher concentration, with elevated levels of CD81 and CD9, which promote enhanced neurite growth and branching, indicating superior regenerative potential [187]. In Alzheimer’s disease models, 3D-cultured MSC-derived EVs displayed distinct miRNA and protein profiles compared to 2D-EVs, highlighting their therapeutic relevance [189]. Similarly, gastric cancer cells cultured in 3D secreted a greater number of smaller EVs, exhibited reduced ADP-ribosylation factor 6, and showed higher microRNA expression than those in 2D culture [190].

A research team demonstrated that EVs derived from MSCs cultured in 3D systems exhibited significantly greater cardioprotective effects in myocardial infarction models compared to those from conventional 2D cultures, underscoring the enhanced therapeutic potential of 3D-EVs [180]. A 3D-cultured BM-MSCs secretomes also promote corneal healing, suggesting that 3D environments support a more favorable niche for producing potent EVs, particularly beneficial for conditions like acute myocardial infarction [184].

In traumatic brain injury (TBI) models, the activity of 3D-cultured MSCs is superior to that shown by 2D-derived MSCs in promoting angiogenesis and neurological recovery [187,191]. This 3D-MSC-associated robust activity is attributed to the physiologically relevant architecture of 3D cultures, which produces EVs with cargo profiles closer to those found in vivo [192].

**Figure 5:**
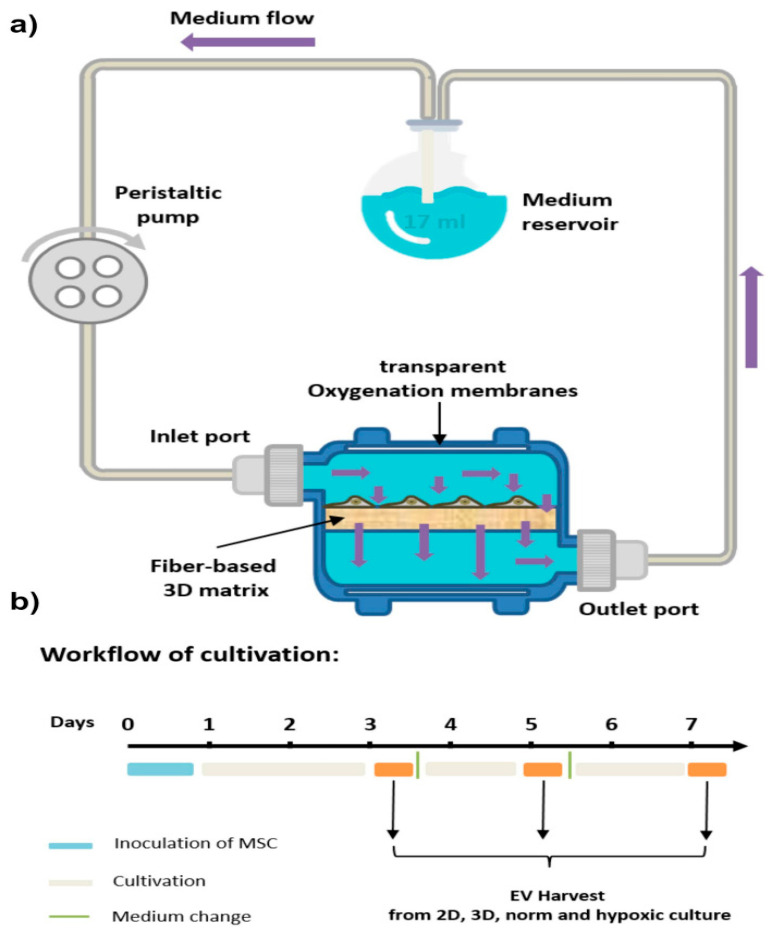
Schematic overview of the 3D cell culture setup. (**a**) The VITVO^®^ 3D bioreactor was integrated with a perfusion pump to establish dynamic culture conditions for mesenchymal stem/stromal cells (MSCs). A flow rate of 1 mL/min was maintained to ensure continuous nutrient supply and efficient waste removal. (**b**) Illustration of the cell culture workflow used for MSC expansion, extracellular vesicle (EV) production, and subsequent EV collection. This figure is reproduced from ref. [193]. © The Author(s) 2024. This article is licensed under a Creative Commons Attribution 4.0 International License, which permits use, sharing, adaptation, distribution, and reproduction in any medium or format, as long as you give appropriate credit to the original author(s) and the source, provide a link to the Creative Commons license, and indicate if changes were made. To view a copy of this license, visit http://creativecommons.org/licenses/by/4.0/.

Culturing MSCs in 3D systems, such as bioreactors or fiber scaffolds, markedly increases the yield and purity of EVs compared to 2D cultures (Figure 5) [193]. A preclinical study demonstrated that systemic administration of hMSC-derived EVs, generated under either 2D or 3D culture conditions, significantly promoted functional recovery in a rat model of TBI [191]. Notably, EVs derived from 3D scaffold cultures exhibited superior benefits, including enhanced spatial learning, increased hippocampal neurogenesis, and greater suppression of neuroinflammation, compared to 2D-EV.

Collectively, extensive research has demonstrated the superiority of 3D culture platforms in producing higher yields of EVs with significantly greater therapeutic potential compared to 2D culture techniques. However, for the clinical application of 3D-EVs, it is crucial to optimize MSC culture conditions to maximize EV yield and improve their therapeutic efficacy [194,195]. Unlike standard 3D cultures, organoids enhance cell diversity and intercellular communication, suggesting that OEVs may possess greater functional complexity than 3D-EVs. The following sections will delve into the production of EVs within these advanced 3D cellular structures, known as organoids. Table 2 summarizes the key differences between EVs derived from 2D cultures and those from 3D/organoid cultures.

## 4. Organoid-Derived EVs (OEVs)

### 4.1. Background

OEVs discussed here are distinct from conventional EVs produced in standard 3D cultures. These OEVs originate from organoids that undergo self-organization and differentiation, forming complex structures composed of multiple specialized cell types. This structural and cellular complexity suggests that OEVs may offer enhanced yield and improved functional properties compared to EVs from general 3D cultures. Supporting this notion, 3D cultures are already known to provide advantages over 2D systems by increasing cell–cell contact and promoting intercellular communication. Organoids go a step further by fostering even more diverse and dynamic cellular interactions, which likely contribute to a greater molecular and functional complexity in OEVs compared to traditional 3D-EVs [65].

Although OEVs and conventional EVs fall under the category of mammalian EVs sharing similar origins, biogenetic pathways, molecular profiles, and mechanisms of cellular uptake, they differ in several key aspects. These differences arise primarily from the unique 3D architecture of organoids and their diverse cellular composition. Notably, OEVs more closely mimic EVs found in human body fluids [65].

### 4.2. OEV Isolation and Characterization Methods

**Figure 6:**
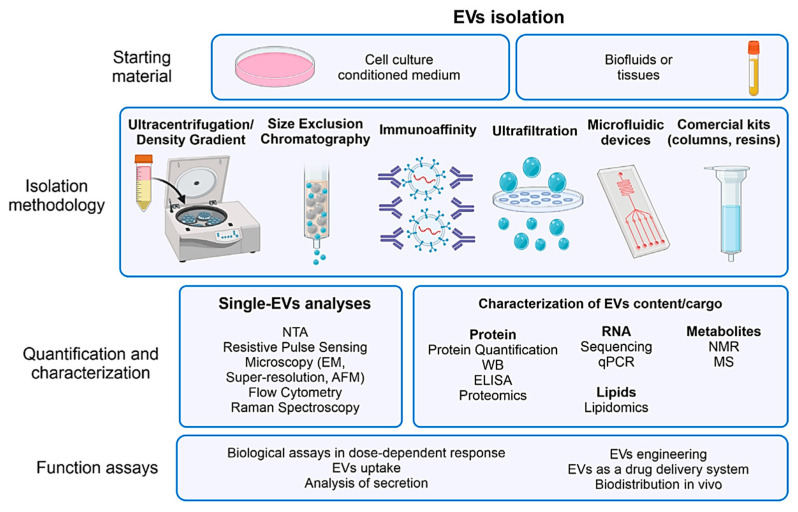
A representative diagram outlining the standard procedures for extracellular vesicle isolation and characterization, highlighting commonly employed techniques at each stage. This figure is reproduced from ref. [55]. © 2022 The Authors. WIREs Nanomedicine and Nanobiotechnology published by Wiley Periodicals LLC. This is an open access article under the terms of the http://creativecommons.org/licenses/by/4.0/ License, which permits use, distribution and reproduction in any medium, provided the original work is properly cited.

Various methods are employed to isolate OEVs, including size-exclusion chromatography, immunoaffinity capture, microfluidic technologies, ultrafiltration, sucrose density gradient centrifugation, ultracentrifugation, and commercial kits (e.g., resins and columns) (Figure 6) [55,65]. Each technique presents unique advantages and limitations depending on the application. Gradient ultrafast centrifugation, based on differences in settlement coefficients, offers high purity and enables the separation of subgroups; however, it is time-consuming and requires advanced equipment [52].

Size exclusion chromatography, which separates particles by size, provides high purity and fast preparation; however, it is costly and yields low output [208]. Immunoaffinity capture utilizes specific ligand binding to isolate EVs, producing highly pure and specific exosomes; however, it is expensive, has low yield, and requires ligand optimization [209]. Microfluidic technology, which combines immunoaffinity with particle size and density differentiation, enables high efficiency and avoids chemical contamination; however, it is limited by low yield and high cost [210]. Sucrose density gradient centrifugation, which relies on centrifugal force, yields high-purity EVs but involves a lengthy, labor-intensive process with low yield [211]. To ensure the high-purity isolation of EVs, it is essential to use culture media free from external EVs, which is typically achieved by supplementing EV-depleted serum to minimize contamination [212,213,214]. Choosing the optimal OEV isolation method involves balancing purity, yield, cost, and processing time to meet the requirements of research or clinical applications.

Following the isolation of OEVs, characterization is typically performed using transmission electron microscopy (TEM), nanoparticle tracking analysis (NTA), Western blotting, electron cryo-electron microscopy, atomic force microscopy, resistive pulse sensing, flow cytometry, and Raman spectroscopy [55,215,216,217,218]. EVs are difficult to detect by flow cytometry due to their small size, low refractive index, and heterogeneity. Detection is further hindered by interference from similar sized lipoproteins and protein aggregates, as well as their low antigen density, which limits the sensitivity of ELISA and Western blotting. TEM and NTA are commonly employed to assess the size distribution, morphology, and concentration of OEVs. Meanwhile, Western blotting helps identify specific surface markers, such as tumor susceptibility gene 101, CD81, CD9, and CD63, which are characteristic of EVs [66,219].

The intricate architecture and cellular diversity of organoids present greater challenges in characterizing OEVs than those encountered with conventional EVs. Standard techniques such as TEM and NTA remain valuable for evaluating vesicle morphology, size distribution, and concentration. However, due to the heterogeneous composition of OEVs, more rigorous and nuanced analytical approaches are necessary.

Vesicle isolation methods, including Western blotting and fluorescent labeling of surface markers, should be tailored to reflect the specific cellular origins of OEVs, ensuring precise and meaningful characterization [220]. Additionally, utilizing ECM-based scaffolds, such as Matrigel, in organoid cultures complicates the isolation of OEVs, making the process more challenging than standard EV isolation. Thus, specialized and meticulous techniques are required to isolate and purify OEVs, preserving their structural integrity and biological functionality while minimizing contamination. Table 3 summarizes the key aspects of EV classification, including representative markers, biogenetic origins, size ranges, recommended usage, and defining features, providing a comprehensive reference for EV research in line with MISEV2023 [54].

In summary, as organoid-based research advances and the functional potential of OEVs becomes more widely recognized, establishing standardized protocols for their isolation and characterization will become crucial.

### 4.3. OEVs Sources and Applications

#### 4.3.1. Ophthalmic OEVs

A research report analyzed EVs derived from hiPSC-based retinal organoids cultured in a PBS Vertical Wheel (PBS-VW) bioreactor and compared them with EVs derived from hUCMSCs [221]. Using atomic force microscopy (AFM), the researcher demonstrated that OEVs possess increased vertical dimensions and enhanced surface roughness. In addition, nanomechanical profiling indicated that OEVs were more pliable, exhibited lower adhesion, and were more easily deformed than hUCMSC-EVs. Western blot analysis demonstrated that EVs from late-stage retinal organoids (Day 200) exhibited increased expression of key exosomal markers, including Alix, HRS, Caveolin-1, CD63, HSP70, and Flotillin-2 [221]. Notably, the use of the PBS-VW bioreactor significantly improved EV yield, enhancing their potential for therapeutic applications.

**Figure 7:**
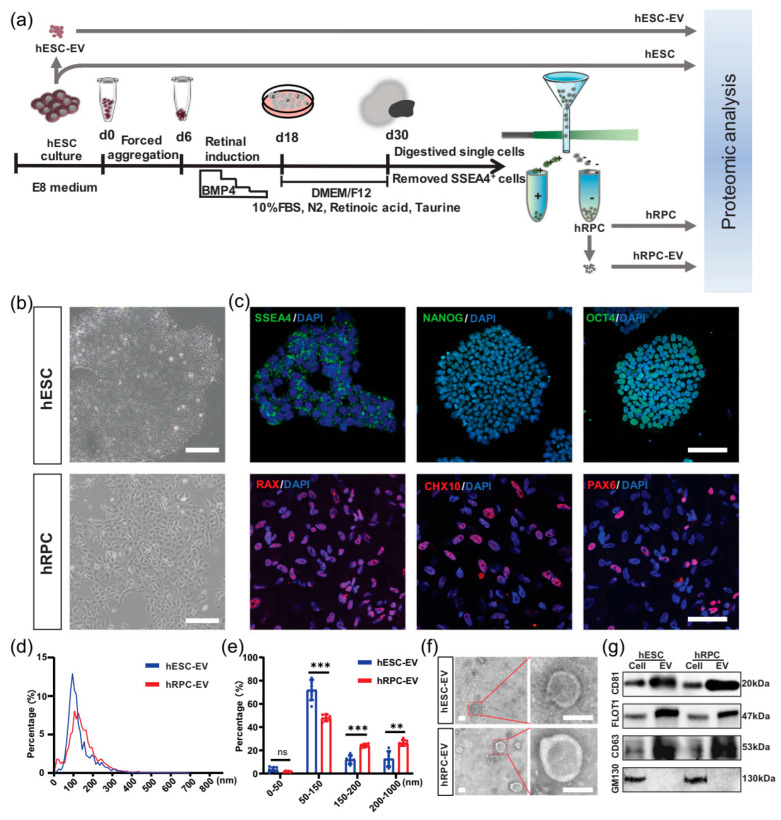
Isolation and characterization of extracellular vesicles (EVs) from human embryonic stem cells (hESCs) and retinal organoid-derived retinal progenitor cells (hRPCs). (**a**) Schematic overview of the experimental workflow for retinal organoid generation and EV analysis. (**b**) Representative light microscopy images showing the morphology of hESCs and hRPCs. (**c**) Immunofluorescent staining confirming the expression of pluripotency markers (SSEA4, NANOG, OCT4) in hESCs and retinal progenitor markers (RAX, CHX10, PAX6) in hRPCs. (**d**,**e**) Nanoparticle tracking analysis showing the size distribution and relative abundance of EVs derived from hESCs and hRPCs. (**f**) Transmission electron microscopy images depicting the ultrastructure of isolated EVs from both cell types. (**g**) Western blot analysis verifying the presence of EV-specific markers in hESCs, hRPCs, and their corresponding EV samples. Data are shown as mean ± SD (*n* = 6 for panel (**e**)). Statistical significance was determined using Benjamini–Hochberg corrected *t*-tests: ** *p* < 0.01, *** *p* < 0.001, ns = not significant. Scale bars: 200 μm (**b**), 100 μm (**c**), 100 nm (**f**). This figure reproduced from ref. [212]. © 2023 The Authors. Journal of Extracellular Vesicles published by Wiley Periodicals LLC on behalf of International Society for Extracellular Vesicles. This is an open access article under the terms of the http://creativecommons.org/licenses/by/4.0/ License, which permits use, distribution and reproduction in any medium, provided the original work is properly cited.

By comparing them with hESC-derived EVs, a research team investigated the properties and potential therapeutic benefits of OEVs obtained from human retinal progenitor cells (hRPC-EVs) for retinal degeneration (RD) (Figure 7) [212]. In vitro experiments demonstrated that OEVs improved phagocytic activity, reduced lipid formation, and mitigated oxidative stress and lipotoxicity in ARPE-19 retinal pigment epithelial cells treated with oleic acid [212]. The integration of hRPC-EVs into the mitochondrial network impacted fatty acid metabolism. Additionally, proteomic analysis demonstrated that OEVs were rich in components related to lipid metabolism, immune modulation, and retinal development.

A study investigated the potential of small EVs (sEVs) derived from human ESC-derived retinal organoids (hERO-RPCs) as a novel therapy for retinal degenerative diseases (RDD) [222]. These sEVS delayed photoreceptor degeneration and preserved retinal function in Royal College of Surgeons (RCS) rats, a model for inherited retinal degeneration. The sEVs were taken up by Müller cells both in vivo and in vitro, where they suppress gliosis and promote early dedifferentiation. The study highlights the therapeutic potential of hERO-RPC-sEVs for retinal degenerative diseases (RDD), demonstrating their ability to protect retinal structure and function [222]. Mechanistically, these effects are mediated by the transfer of specific miRNAs, particularly miR-21-5p and miR-92a-3p, which downregulate nuclear factor I B (NFIB), thereby altering the gliotic fate of Müller cells. These findings provide novel insights into stem cell-based therapies and support the advancement of EV-centered treatment strategies for RDD.

Another research team demonstrated the therapeutic potential of exosomes derived from retinal organoids (Exo-ROs) for treating retinal degenerative diseases (RD) [223]. In the RCS rat model, Exo-ROs delayed photoreceptor loss and preserved visual function by reducing photoreceptor apoptosis and preventing thinning of the outer nuclear layer (ONL).

These protective effects were primarily mediated by exosomal microRNAs (miRNAs) targeting the mitogen-activated protein kinase (MAPK) signaling pathway. Intravitreal administration of Exo-ROs effectively mitigated photoreceptor cell death, maintained retinal structure, and restored visual function [223]. Notably, this non-cellular therapeutic approach circumvents the safety risks associated with direct cell transplantation, positioning Exo-ROs as a promising platform for developing therapies for retinal degeneration.

A separate study comprehensively characterized 3D human retinal OEVs cultured in bioreactors, aiming to explore their potential in ocular therapeutics, biomarkers, and drug delivery [221]. Compared to EVs from human umbilical cord MSs, retinal organoid EVs, particularly from mature organoids (>120 days), showed elevated expression of exosome biogenesis genes (e.g., TSG101, ALIX, RABs, CD63, CD81) and exosomal protein markers (HRS, Caveolin-1, HSP70, Flotillin-2). These EVs exhibited distinct biophysical and nanomechanical properties, with enhanced biogenesis and a cargo of proteins related to retinal function [221]. The study highlights retinal organoid-derived EVs as a promising, scalable source for therapeutic and diagnostic applications in ocular diseases such as AMD, RP, glaucoma, and diabetic retinopathy.

Park et al. investigated exosomal miRNAs as non-invasive biomarkers for assessing the quality of differentiation in human retinal organoids (ROs) [224]. They found that well-differentiated (superior) ROs secrete a broader diversity of exosomal miRNAs than poorly differentiated (inferior) ones. Differentially expressed miRNAs in superior ROs targeted genes involved in key biological pathways, such as neuronal proliferation, oxidative stress response, mRNA metabolism, and DNA damage repair, particularly through the FoxO and p53 signaling pathways.

Notably, upregulated miRNAs were linked to neuroprotection, while downregulated ones were associated with mRNA turnover and regulation of senescence [224]. This study highlights hsa-miR-654-3p and hsa-miR-451a as promising biomarkers for non-invasive quality control of RO differentiation, offering a potential tool for improving reproducibility, scalability, and standardization in retinal organoid production for research and therapeutic applications.

#### 4.3.2. Skin OEVs

A group of scientists has efficiently developed epidermal organoids (iEpiOs) from iPSCs, which exhibit stratified architecture mimicking the natural layers of the epidermis, including the basal, spinous, granular, and cornified layers [225]. In contrast, culturing iEpiOs on a 2D monolayer platform resulted in the loss of typical organoid features. The researcher then isolated EVs from both 2D and 3D cultured organoids and compared their yield and characteristics. Nanoparticle tracking analysis (NTA) revealed that EV yield under 3D conditions was approximately double that of the 2D cultures, while the particle size remained comparable. EVs derived from 3D cultures showed increased expression of CD9, but reduced levels of Alix and Tsg101. Notably, these 3D-EVs were enriched in vascular endothelial growth factor (VEGF) [225].

Further assays, including PCR and tube formation, demonstrated that 3D-EVs significantly enhanced VEGF mRNA expression in human umbilical vein endothelial cells (HUVECs) and promoted angiogenesis. Small RNA sequencing (sRNA-seq) identified 16 miRNAs significantly upregulated in 3D-EVs, which predominantly targeted genes involved in cell growth, migration, tissue development, epithelial differentiation, and angiogenesis. In vitro assays have demonstrated that 3D-EVs stimulate fibroblast growth and mobility, as well as enhance the formation of new blood vessels by endothelial cells, effects that are partly attributed to miR-146a-5p and miR-31-5p [225].

**Figure 8:**
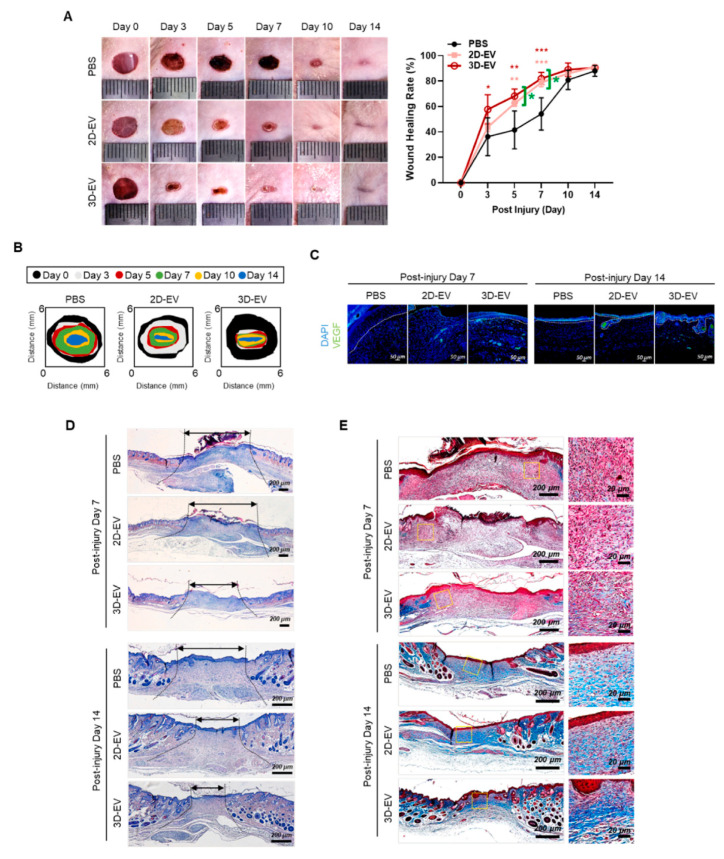
In vivo wound healing of activity of 2D- and 3D-EVs. (**A**) Phase contrast microscope image depicting the progression of full-thickness skin wound healing in mice treated with PBS (control), 2D-EVs (100 μg), or 3D-EVs (100 μg) at days 0, 3, 5, 7, 10, and 14 post-injury. Wound closure rates were quantified using ImageJ (*n* = 6) and expressed as mean ± SEM (* *p* < 0.05; ** *p* < 0.01; *** *p* < 0.001). (**B**) A diagrammatic illustration tracing the wound closure over time. (**C**) Immunofluorescence images displaying VEGF expressions to assess angiogenesis within the wound site. Dashed lines demarcate the epidermal and dermal layers. (Scale bar: 50 μm) (**D**) H&E-stained sections of wound tissue on days 7 and 14 highlight healing dynamics; black arrows indicate scar margins. (Scale bar: 200 μm). (**E**) Masson’s trichrome staining reveals collagen organization and maturity across groups at days 7 and 14. (Scale bars: 200 μm and 20 μm). This figure is reproduced from ref. [225] with permission. © 2024 The Authors. Published by Elsevier Ltd. This article is available under the https://creativecommons.org/licenses/ license and permits non-commercial use of the work as published, without adaptation or alteration provided the work is fully attributed.

These functional advantages were validated in a mouse skin wound healing model, where treatment with 3D-EVs led to a 1.6-fold increase in wound closure on days 3, 5, and 7 compared to controls. Immunohistochemical analysis further confirmed elevated VEGF expression at the treatment sites (Figure 8). This study highlights a key observation: when organoids are transitioned from 3D to 2D cultures, they lose spatial architecture and key organoid traits. This structural change corresponds with distinct differences in marker expression and the biological properties of secreted EVs.

#### 4.3.3. Salivary Gland-Derived EVs

**Figure 9:**
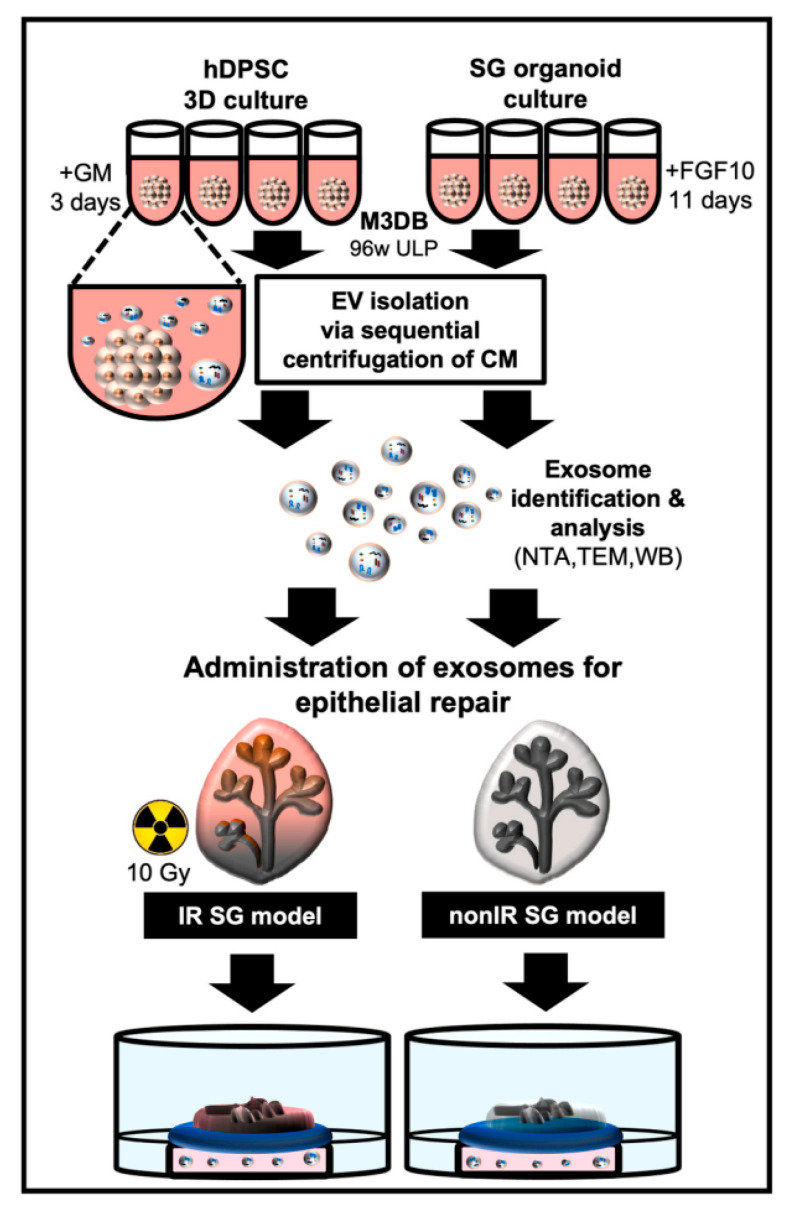
Schematic diagram illustrating exosome-based strategies for salivary gland (SG) epithelial repair using 3D cultures of human dental pulp stem cells (hDPSCs) and SG organoids through utilizing magnetic 3D bioassembly (M3DB). hDPSCs were first subjected to magnetization with nanoparticles and assembled in a 96-well ultra-low attachment plate using a magnetic drive. SG organoids were then differentiated in FGF10-enriched medium, while hDPSCs remained in standard growth medium. Exosomes were isolated from conditioned media using sequential centrifugation. These exosomes, derived from both hDPSC and SG organoids, were incorporated into SG culture media and applied to irradiated (IR) and non-irradiated (non-IR) SG models to assess epithelial repair. This figure is reproduced from ref. [214]. © 2022 The Authors. This is an open access article under the CC BY license (http://creativecommons.org/licenses/by/4.0/).

Chansaenroj et al. developed a novel non-invasive platform, utilizing magnetic 3D bioassembly (M3DB), for the 3D culture of magnetic nanoparticle-labeled human dental pulp stem cells (hDPSCs) for the ultimate generation of highly functional salivary gland organoids (SGo) (Figure 9) [214]. The researchers demonstrated the strong expression level of salivary gland-associated markers (KRT5, KRT14, AQP1, TUBB3, MUC7, and CHRM3) in the developed organoids. Subsequently, EVs derived from both 3D-cultured hDPSCs and SGo referred to as 3D-hDPSC-EVs and SGo-OEVs, respectively, were isolated and assessed in an epithelial injury model.

Notably, the research team demonstrated that the therapeutic outcomes of 3D-hDPSC-EVs were 15%, while the direct transplantation of SG organoids showed 25% therapeutic improvement, and SGo-OEVs demonstrated up to 60% in enhancing epithelial growth. Proteomic profiling of SGo-OEVs revealed the altered expression of 99 proteins, many of which are associated with cell growth, differentiation, adhesion, and regulatory pathways.

These findings highlight the superior biological activity of OEVs compared to conventional 3D-EVs [214]. Remarkably, the regenerative effect of OEVs even surpassed that of direct organoid transplantation, raising the possibility that OEVs may offer a more effective therapeutic modality. Further studies are warranted to determine whether this enhanced efficacy is unique or broadly applicable across other organoid models.

#### 4.3.4. Brain OEVs

Liu et al. explored the labeling of human forebrain OEVs with ultrasmall superparamagnetic iron oxide nanoparticles (USPIOs) for in vitro MRI tracking [226]. Given the therapeutic promise of stem cell-derived EVs in neurological diseases, their clinical use is hindered by limited targeting and efficacy post-injection. This study showed that sonication-based USPIO labeling enables MRI detection without significantly altering EV size, though it reduced EV yield and miRNA content [226]. These results support further in vivo studies while highlighting the need to optimize labeling protocols to preserve EV integrity.

Cerebral OEVs have demonstrated neuroprotective effects comparable to those of MSC-EVs, as reported in a previous study [227]. In the rat midbrain, both EV types reduced H_2_O_2_ treatment-mediated oxidative stress and apoptosis. Owing to their rich content of neurotrophic factors such as neurotrophin-4 (NT-4) and glial cell line-derived neurotrophic factor (GDNF), OEVs demonstrated superior efficacy over MSC-derived EVs in promoting the differentiation of hiPSCs into dopaminergic (DA) neurons [227].

A recent study highlighted the potential of human cerebral organoids as an in vitro model for exploring EVs and their associated miRNAs in neurotoxicity and neurodegenerative diseases [228]. These organoids consistently released small EVs (mean size ~100 nm), displaying typical cup-shaped morphology and expressing canonical EV markers (CD63, TSG101) while lacking mitochondrial contamination (VDAC) [228]. KEGG analysis of EV-associated miRNA targets revealed enrichment in neurological disease pathways, including Alzheimer’s, Parkinson’s, and Huntington’s diseases. IPA analysis further linked EV miRNAs to cellular stress, neurodevelopment, apoptosis, and neurotoxicity pathways. These findings support cerebral organoids as a promising platform for identifying EV-based biomarkers in the early stages of neurodegeneration and neurotoxicant exposure.

#### 4.3.5. Intestinal OEVs

Zhang et al. extracted intestinal crypt OEVs and demonstrated their role in modulating immune responses and maintaining gut homeostasis [229]. These vesicles inhibited cytokine production triggered by lipopolysaccharide (LPS) in various immune cells and promoted communication between enterocytes and immune cells, thereby supporting a balance between bacterial tolerance and immune defense.

**Figure 10:**
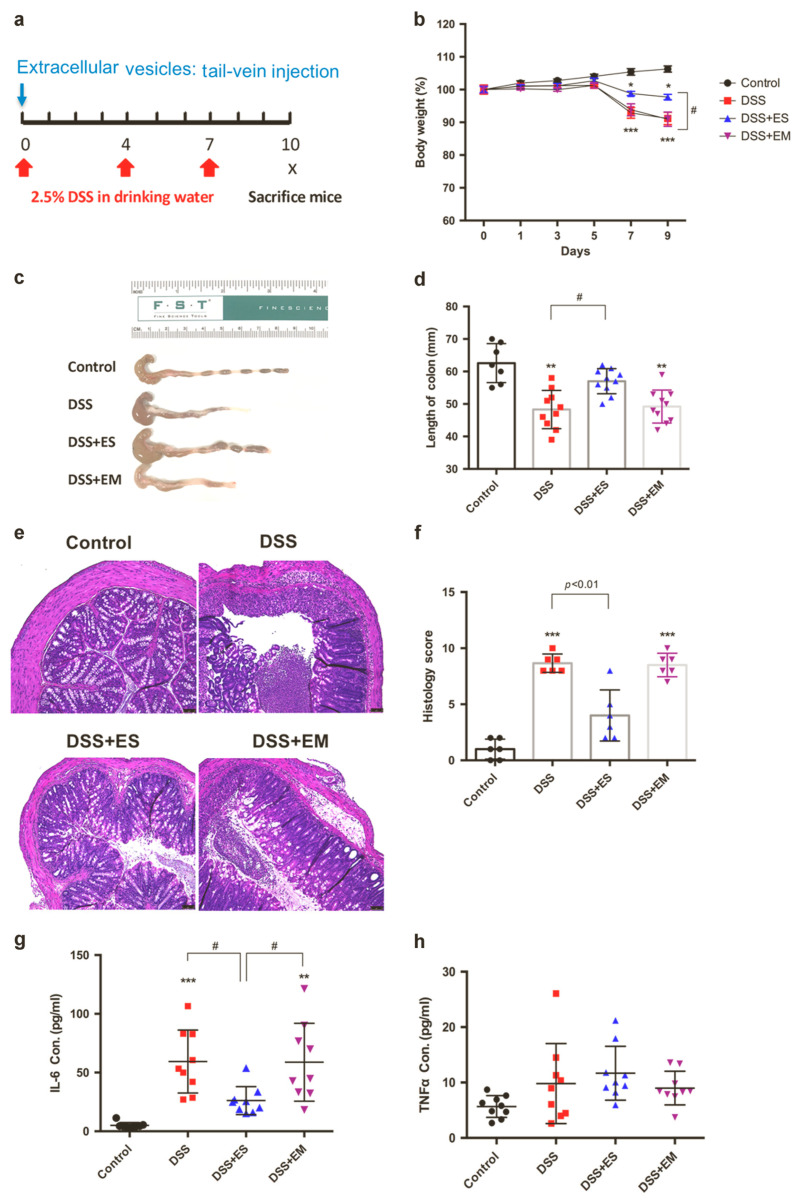
The in vivo therapeutic activity of extracellular vesicles (EVs) from saline-treated intestinal organoids (ES) in mitigating Dextran Sulfate Sodium (DSS) in mice, in contrast to the activity shown by EVs from morphine-treated organoids (EM). (**a**) Schematic of the experimental timeline. (**b**) Body weight changes across treatment groups. (**c**,**d**) Colon length measurements revealed significant differences following EV injection. (**e**) Representative H&E-stained colon tissue images from control and DSS groups (Scale bar: 50 µm). (**f**) Histological scoring was conducted based on standard criteria for colitis. (**g**,**h**) ELISA results showed levels of IL-6 and TNF-α in plasma, with each point representing an individual mouse (*n* = 6–10/group; mean ± SEM; * *p* < 0.05; ** *p* < 0.01; *** *p* < 0.001; # *p* < 0.05). This figure is reproduced from ref. [229] with permission. Copyright © 2021 © Society for Mucosal Immunology. Published by Elsevier Inc. All rights are reserved, including those for text and data mining, AI training, and similar technologies.

EVs from saline-treated intestinal organoids (EV(Org/S)) significantly suppressed LPS-induced pro-inflammatory cytokines and elevated anti-inflammatory cytokines in various immune cells, including mouse BMDCs, BMMs, microglia, and human monocytes. In contrast, EVs from morphine-treated organoids (EV(Org/M)) failed to exert these effects. Let7c-5p was identified as a key mediator, directly targeting IL-6 and mediating the anti-inflammatory properties of EV(Org/S); its inhibition nullified the EVs’ protective effects. In vivo, EV(Org/S) reduced systemic inflammation in an LPS-induced sepsis model and alleviated DSS-induced colitis, while EV(Org/M) showed no such benefits (Figure 10). Notably, Let7c-5p levels were markedly decreased in Dextran Sulfate Sodium (DSS)-treated mice and were further suppressed by morphine administration [229]. The potent immunomodulatory activity of OEVs is largely driven by miRNAs from the Let-7 family, especially Let-7c-5p. Remarkably, morphine markedly reduced Let-7c-5p levels in OEVs, diminishing their anti-inflammatory function and potentially exacerbating inflammatory conditions such as sepsis and colitis.

In sum, this study demonstrated that intestinal crypt organoid-derived EVs are crucial for maintaining mucosal homeostasis by regulating immune responses through the delivery of specific miRNAs. Notably, morphine treatment disrupts this regulatory mechanism by impairing the incorporation of key miRNAs, particularly Let-7c-5p, into EVs. The resulting reduction in EV-associated Let-7 exacerbates immune dysregulation, leading to heightened inflammation in conditions such as sepsis and colitis. These findings suggest that intestinal organoid-derived EVs hold promise as therapeutic or diagnostic tools for inflammatory diseases and identify opioid use as a critical risk factor for inflammation exacerbation.

#### 4.3.6. Tumor Organoid-Derived EVs

Schuster et al. developed a 3D glioblastoma (GBM) organoid model to better replicate the in vivo tumor microenvironment of this aggressive brain cancer [230]. Their findings revealed that 3D organoid cultures secreted significantly more EVs with distinct cargo compared to conventional 2D cultures. Notably, EVs derived from 3D models exhibited enriched and differentially expressed miRNAs, including upregulation of pro-invasive miR-23a-3p and downregulation of tumor-suppressive miR-7-5p. These EVs were associated with immune-related pathways such as IL-4, IL-13, and IL-12 signaling, highlighting the influence of 3D culture conditions on tumor microenvironment (TME) modulation and EV-mediated communication.

Four miRNAs (miR-323a-3p, miR-382-5p, miR-370-3p, and miR-134-5p) within the cancer-associated DLK1-DIO3 domain were found to be regulated in 3D glioblastoma cultures, suggesting their relevance in tumor progression [230]. EVs derived from 3D organoids exhibited alterations in immune regulatory pathways and aggressive phenotypes, such as downregulated miR-7-5p, indicating a closer resemblance to in vivo glioblastoma conditions than 2D cultures. These findings highlight the potential of 3D glioblastoma models for biomarker discovery and therapeutic development, particularly in targeting the immunosuppressive tumor microenvironment [230]. Notwithstanding the heterogeneity of GBM, the consistent enrichment of immune-related EV signatures supports the value of 3D platforms and encourages further investigation using cerebrospinal fluid liquid biopsies.

A research team demonstrated that 3D cell culture using NanoCulture Plates (NCPs) significantly influenced cancer cell aggregation, stem-like properties, and EV secretion [231]. Among the 67 cell lines analyzed, grape-like aggregation (GLA) emerged as a distinct and aggressive characteristic, predominantly observed in adenocarcinoma-derived cells. Notably, the neuroendocrine adenocarcinoma cell line PC-3, which exhibited GLA, formed rapidly growing, asymmetric, and lymph node–node-metastasizing tumors in immunocompromised mice, unlike its spheroid- or monolayer-forming counterparts, indicating improved in vivo relevance.

Culturing in stem cell medium within the NCP-based 3D nanoenvironment promoted the development of large, slowly growing organoids that expressed stem cell markers, neuroendocrine markers, adhesion molecules, and oncogenes, along with the formation of a hypoxic core that closely mimicked in vivo tumors [231]. This system offers a physiologically relevant model for investigating tumor biology, cancer stem cells, and preclinical therapeutics. Furthermore, the elevated release of EpCAM- and CD9-positive exosomes, as well as HSP90, from these CSC-like aggregates underscores their potential as diagnostic or therapeutic targets.

A research work performed by Nagai et al. aimed to identify miRNA signatures in exosomes derived from patient-derived organoids of human colorectal adenoma (CRA) and colorectal cancer (CRC) [232]. Researchers successfully established eight CRA and six CRC organoid lines that closely recapitulated the original tumor architecture. Exosomes isolated from conditioned media were confirmed by transmission electron microscopy to be ~100 nm in diameter, with a mode size of 103.5 ± 3.2 nm, and showed positive expression of exosomal markers CD9 and CD63 [232]. This work is the first to report distinct exosomal miRNA profiles in CRA- and CRC-derived organoids, notably identifying significant upregulation of miR-1246 in CRC organoids. Functional assays using miR-1246 mimics and inhibitors in HT-29 CRC cells revealed that miR-1246 promotes cell proliferation, suggesting a potential role in the CRA-to-CRC transition.

Additionally, this study assessed the feasibility of using 3D organoid-derived exosomes to explore tumor biology and highlights miR-1246 as a candidate biomarker or therapeutic target [232]. There are limitations, such as the exclusion of metastatic samples, the uncertain cellular origins of exosomal miR-1246 within heterogeneous organoids, and the need for further mechanistic insights into its role in CRC progression.

A recent proteomic study investigated EVs derived from human pancreatic ductal adenocarcinoma (PDAC) organoids and healthy control (HC) pancreatic organoids to identify distinct EV protein signatures for early disease detection and to elucidate changes in cellular programming during tumor progression [233].

PDAC organoid-derived EVs exhibited markedly different protein profiles compared to those from healthy organoids. Tumor-derived EVs were enriched in proteins associated with vesicular transport and tumorigenesis, including Laminin subunit alpha 5 (LAMA5), Syntenin-1 (SDCBP), and Tenascin (TENA), all of which are implicated in cancer progression, metastasis, and exosome biogenesis. In contrast, EVs from healthy organoids predominantly contained proteins associated with cellular homeostasis. Tetraspanin markers (CD9, CD63, CD81, and Tspan8) were detected in EVs from both groups, although Tspan8 was more consistently detected than CD81 [233]. These findings highlight the potential of organoid-derived EV proteomics to differentiate malignant from non-malignant states and underscore the utility of EV protein markers as a basis for developing multiplexed plasma-based screening tools for early pancreatic cancer diagnosis and monitoring.

Huang et al. demonstrated the potential of patient-derived xenograft (PDX)-derived organoids (PXOs) as a translational model for pancreatic ductal adenocarcinoma (PDAC) research [234]. By analyzing conditioned media from tumor organoids, they developed a biomarker discovery pipeline and identified four EV proteins, including ANXA11, CD44v6, CD14, and GPC4, as candidate circulating biomarkers. ANXA11 emerged as the most promising marker, showing significant enrichment in PDAC patient plasma compared to plasma from patients with benign gastrointestinal diseases [234]. Mass spectrometry analysis revealed 241 tumor-enriched EV proteins, and principal components analysis (PCA) distinguished PDAC from other tumor types. This study establishes PXOs as a scalable and cost-effective platform for discovering clinically relevant biomarkers; however, further validation is required to confirm their diagnostic utility.

## 5. Future Perspectives

Traditional 2D cultures limit EV production due to restricted surface area and reduced cell–cell interaction, which can affect EV quality. In contrast, 3D systems enable efficient expansion of cell populations, better maintain cellular characteristics, and enhance EV yield, release duration, and retention [204].

The 3D culture environment enhances key cellular signaling, supporting stemness, survival, and regeneration [183]. Stem cells grown in 3D also release more anti-inflammatory and pro-angiogenic factors, boosting tissue repair [235]. These benefits make 3D platforms more effective than traditional 2D systems for stem cell therapy and regenerative medicine.

Organoid culture platforms are a promising bridge between animal models and clinical trials, enabling personalized medicine through patient-derived models [30]. Their scalability supports high-throughput drug screening and long-term evaluation of drug efficacy and toxicity [236,237]. Additionally, autologous organoid transplantation may reduce immune rejection and support tissue regeneration, paving the way for future therapies in cancer, organ failure, and wound repair.

Recent studies have highlighted the therapeutic, diagnostic, and drug delivery potential of EVs; however, their limited yield remains a major barrier to clinical use, driving efforts to boost EV production [204]. The 3D stem cell culture offers significant advantages over the 2D culture, as it more accurately replicates the native microenvironment of cells. Three-dimensional culture systems enhance cell–cell and cell–matrix interactions, which are crucial for maintaining cellular integrity, differentiation potential, and functional activity [61,238].

OEVs are nanoscale lipid particles secreted by 3D organoids that better replicate the native tissue environment compared to traditional EVs. These vesicles carry biomolecules that modulate gene expression in target cells and offer advantages such as low immunogenicity, efficient cargo delivery, and reduced risks compared to cell-based therapies [65]. OEVs also show enhanced production, physiological relevance, tissue specificity, and molecular complexity, making them promising tools for therapeutic applications [239].

Although the robust therapeutic potential of OEVs is recognized, their clinical translation faces several hurdles [240]. Key challenges include the lack of standardized protocols for isolation, characterization, and large-scale, GMP-compliant production. The quality and cellular composition of organoids, often missing stromal, immune, or vascular elements, can limit OEV functionality and the accuracy of disease modeling. Incorporating stromal cells, such as fibroblasts or MSCs, to promote extracellular matrix remodeling and structural organization is essential when designing future strategies [241]. The addition of immune cells, including macrophages, dendritic cells, and T cells, has been shown to enhance the modeling of inflammation, immune surveillance, and tumor–immune interactions [242,243]. To overcome the limitations of nutrient diffusion and hypoxia, vascularization strategies are being developed, such as embedding endothelial cells into organoid systems [244], utilizing microfluidic “organ-on-a-chip” technologies [245], and applying 3D bioprinting approaches to create perfusable vascular networks [246]. Collectively, these approaches represent critical directions for developing next-generation organoid platforms that more authentically recapitulate native tissue complexity and provide a robust foundation for OEVs research.

Additionally, the culture environment and factors used can alter EV cargo, raising concerns about reproducibility. Isolation becomes even more complex when organoids are grown in matrices, such as hydrogels or ECM. One of the persistent challenges in the field is the isolation of OEVs from complex matrices such as Matrigel. Matrigel’s protein-rich composition, which includes laminin, collagen IV, entactin, and various growth factors, can co-isolate with EVs complicating downstream analyses. These contaminants not only compromise proteomic and lipidomic profiling but may also obscure the identification of vesicle-specific biomarkers and functional cargo, thereby limiting the interpretability and reproducibility of OEV studies.

Several strategies have been explored to address these limitations. Matrix-free organoid culture platforms, including defined synthetic hydrogels or suspension systems, reduce background contamination and improve the purity of recovered OEVs [247]. In parallel, advances in EV isolation methods, such as optimized ultracentrifugation protocols, size-exclusion chromatography (SEC), and iodixanol density gradients, have been coupled with proteomic filtering to discriminate OEV-specific proteins from matrix-derived components [248,249]. Notably, chromatography-based purification (anion exchange or bind-elute approaches) and microfluidic isolation techniques have shown promise for selectively enriching EVs while minimizing carryover of matrix proteins and preserving vesicle integrity [250]. More recently, microfluidic-based isolation platforms have emerged as promising tools, enabling selective enrichment of EVs with reduced Matrigel carryover while preserving vesicle integrity [251].

Despite these advances, achieving high-purity OEV preparation remains a significant challenge, particularly when scaling for translational applications. Future work should prioritize the development of standardized, matrix-free culture systems and orthogonal purification pipelines to ensure that downstream functional and molecular analyses truly reflect the biology of OEVs. Such improvements will be critical for enhancing reproducibility, comparability across studies, and ultimately, the translational potential of OEV-based therapies. Further studies are needed to understand OEV mechanisms and optimize their therapeutic use.

**Figure 11:**
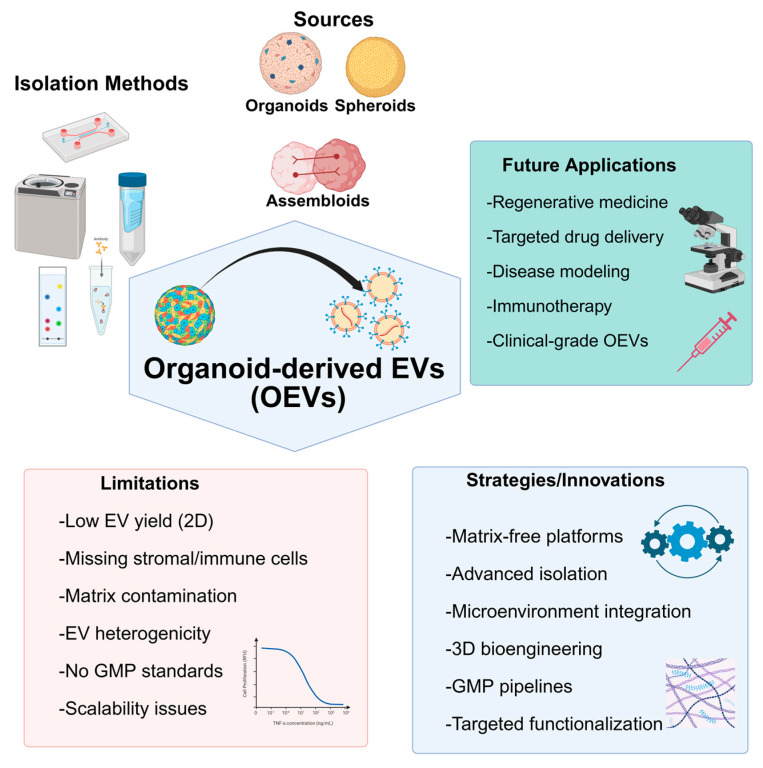
Schematic overview of organoid-derived extracellular vesicles (OEVs): Sources include spheroids, organoids, and assembloids. Common isolation methods involve ultracentrifugation, chromatography, immunoaffinity, microfluidics, and ultrafiltration. Key limitations include low yield in 2D cultures, lack of stromal/vascular/immune components, matrix contamination, cargo variability, absence of GMP-compliant protocols, and scalability issues. Strategies to overcome these challenges encompass matrix-free organoid platforms, advanced isolation methods, microenvironmental integration, 3D bioengineering, GMP-compliant pipelines, and functionalization approaches. Future applications of OEVs include regenerative medicine, precision drug delivery, high-fidelity disease modeling, immunomodulatory therapies, and scalable clinical-grade therapeutics. Created with BioRender.com.

OEVs from retina have demonstrated efficacy in promoting corneal wound repair, highlighting their promise in ocular therapy [221]. Their distinct protein and RNA signatures also offer advantages for biomarker discovery, especially in cancer, due to the organoid system’s superior tissue specificity over traditional 2D or standard 3D cultures [252]. To address current challenges, future studies should focus on developing efficient and reproducible methods, such as microfluidic sorting, for isolating OEVs while maintaining vesicle integrity. Standardizing organoid culture and OEV characterization protocols will reduce variability and enhance reproducibility. Incorporating stromal and immune cells can improve the physiological relevance of organoid systems, and bioengineering strategies may further functionalize OEVs for targeted drug delivery. Overall, overcoming these technical and biological hurdles is essential for advancing the clinical application of OEVs in diagnostics and therapy (Figure 11).

## 6. Conclusions

OEVs hold great promise as next-generation tools for regenerative medicine, disease modeling, and precision therapeutics. Their ability to provide higher yield, greater tissue specificity, and enhanced molecular complexity compared to conventional EVs underscores their translational potential. However, several barriers remain, including the lack of standardized culture and isolation protocols, limited scalability, and incomplete incorporation of key microenvironmental components. Future progress will depend on multidisciplinary strategies that refine culture systems, ensure GMP-compliant production, and advance bioengineering for improved targeting and safety. Once these challenges are addressed, OEVs could emerge as a cornerstone technology for personalized and regenerative healthcare. 

## Figures and Tables

**Table 1:** Comparison of representative bioreactor systems for stem cell and organoid culture.

Manufacturer/ System	Type	Volume Range	Key Features/ Advantages	Limitation	Application	Ref.
Spinner Flask (Corning, Thermo Fisher Scientific)	Stirred	50–500 mL	- High expansion, nutrient mixing. - Simple, scalable; impeller-driven mixing; easy monitoring	High shear, larger volume	- Widely used for embryoid body (EB) formation and initial organoid aggregation	[90,91,93,94]
Stirred-Tank Bioreactor (Eppendorf, DASbox Mini Bioreactor), Sartorius (BIOSTAT^®^)	Mixing mode	10–200 mL	- Mass Production. - Maintain the pluripotency - Controlled mixing, pH, O_2_, and nutrient monitoring. - Scalable for large cultures - Matrix-free	- Low yields - Hydrodynamic stress	Scalable production of cerebral, intestinal, and liver organoids; supports GMP-compatible workflows	[91,94,95,96]
Wave Bioreactor Cytiva (WAVE Bioreactor™)	Rocking-motion bioreactor (Gentle mixing with disposable bags)	25–100 mL	- Scale-up and automation - Low shear stress	- Limited high-volume. - Scaling up beyond 100 L can be challenging - Space Requirement	Expansion and maturation of stem cell–derived organoids; reduced shear stress	[97,98]
Miniaturized spinning bioreactor RPMotion (Orgonex, RPMotion)	Mini Spinning	5–50 mL	- Fast expansion, minimal manual labor - 32-vessel multiplexing; plug-and-play; LCD control; compatible with standard incubators	- Small scale. - Initial setup cost	- Human epithelial organoids (long-term expansion, high proliferation, and reproducibility)	[99]
Rotating Wall Vessel (RWV) (Synthecon, NASA)	Rotating Wall Vessel	10–50 mL	- Simulated microgravity; low shear; optimized for differentiation, enhanced mass transfer - Low mechanical stress, better patterning	Complex design	Promotes 3D aggregation and improved nutrient exchange for neural and hepatic organoids	[89,100]
Microfluidic Bioreactor (Emulate Inc., Mimetas (OrganoPlate^®^), CN Bio)	Microfluidic	0.5–10 mL	- Small channels, high environmental control, high throughput - High content screening, minimal volume. - Superior tissue functionality.	Device fabrication required	- Efficient cell entrapment and spheroid formation. - Generation of liver organoids and retina-on-a-chip	[101,102,103]
Hollow Fiber (FiberCell Systems, Repligen)	- Perfused High surface area-to-volume ratio	20–100 mL	- Controlled shear; high gas/perfusion exchange; cell–cell contact - Controlled gradient, long-term culture	Difficult to operate	- Long-term culture. - Large scale production. - Stem cell differentiation (immune cell, neuronal cells, hepatocytes, osteoblast)	[104,105]

**Table 2:** The comparison between 2D and 3D/Organoids-EVs.

Aspect	2D-EVs	3D-EVs/OEVs	Ref.
Cellular milieu	- Flat, monolayer culture. -Limited cell–cell and cell–matrix communications	- Scaffold-based or scaffold-free platforms. - Support cellular communications - Multicellular - Spatially organized	[8,196]
Mimicry of native tissue	- Poorly recapitulate the native tissue conditions	- High tissue fidelity - Mimics in vivo microenvironment	[185,197]
Cellular complexity	- Simple and single cell type	- Multiple cell types	[65,66]
Yield and cargo content	- Lower yield as per cell and therapeutic cargo loading	- Higher yield. - Enriched in tissue-/ cell-specific miRNAs, proteins	[185,198,199]
Drug screening & disease modeling	- Less predictive of patient-specific responses	- Enables precision modeling for drug testing and pathology	[200,201]
Scalability	- Easily scalable for basic research	- Technically complex - Large-scale production	[202,203,204]
Applications	- Basic EV biology. - Simple therapeutic trials	- Disease modeling, - Tissue regeneration.	[65,205]
Limitations	- Poor tissue mimicry - Low functional cargo	- Standardization, scalability, and cost: Under development	[185,206,207]

**Table 3:** Standardized definitions, markers, and characteristics of extracellular vesicles (EVs) according to International Society for EVs (ISEV) classification.

EV Name	Markers	Biogenesis	Size	Usage Recommendation	Key Features
Extracellular Vesicles (EVs)	Category 1 (membrane-associated): Tetraspanins (CD9, CD63, CD81, CD82), integrins, LAMP1/2, CD73. - Category 2 (cytosolic): ESCRT components (TSG101, ALIX), flotillins, heat shock proteins (HSC70, HSP84), actin, tubulin, GAPDH. - Category 3 (Negative/contamination markers): Calnexin (ER), GM130 (Golgi), Cytochrome C (mitochondria), Histones (nuclei).	Lipid bilayer vesicles released from cells, lacking replication	Nano- to micro-sized particles	Recommended	- Heterogeneous population. - Identification requires multiple complementary methods
Non-vesicular Extracellular Particles (NVEPs)	- Non-vesicular particles: LPPs, RNPs, viruses, exomeres, supermeres. - Purity markers: Apolipoproteins, immunoglobulins, Tamm–Horsfall protein, albumin.	Multimolecular assemblies released from cells that lack a lipid bilayer.	Nanometer to micron size range,	Recommended	- No lipid bilayer - Overlap with EV physicochemical properties
Extracellular Particles (EPs)	No single universal markers.	Biogenesis depends on origin but is often undefined.	Nanometer to micron size range.	Recommended	- Vesicular EPs: Exosomes (MVBs), microvesicles (plasma membrane), apoptotic bodies (cell death). - Non-vesicular EPs: Lipoproteins, RNPs
Small EVs (sEVs)	Category 1 & 2 proteins, but none are universally specific to small sEVs.	Mixed populations: endosomal (exosome) and plasma membrane (ectosome).	<200 nm in diameter.	Recommended, with caution	Includes both exosomes and small ectosomes.
Large EVs (lEVs)	Category 1 & 2 proteins.	Plasma membrane budding/fission.	>200 nm in diameter.	Recommended, with caution	Diameter varies with isolation and characterization method.
Apoptotic bodies	Not specified sources.	Formed during programmed cell death (e.g., apoptotic bodies).	Not specifically defined in the source but recognized as a distinct EV subtype.	Recommended, with caution	Morphology: Typically fragmented and irregular in shape.
Exosome	- LAMP1: Suggested marker. - CD9, CD63, CD81.	- Derived from endosomes. - Released via MVB–plasma membrane fusion.	<200 nm.	-Discouraged unless subcellular origin is clearly defined.	-Subset of sEVs, but not synonymous with all sEVs.
Ectosome (Microvesicle, Microparticle)	Annexin A1, SLC3A2, BSG (specificity uncertain).	- Wide size range - Overlaps with exosomes.	Broad size range.	Discouraged unless origin is clearly defined.	Also referred to as microvesicles/microparticles.

## Data Availability

No new data were created or analyzed in this study. Data sharing is not applicable to this article.

## Abbreviations

The following abbreviations are used in this manuscript:

2D	Two-Dimensional
3D	Three-Dimensional
EVs	Extracellular vesicles
iPSC	Induced pluripotent stem cells
BM-MSCs	Bone marrow-derived mesenchymal stem cells
MVBs	Multivesicular bodies
ILVs	Intraluminal vesicles
ESCRT	Endosomal sorting complex required for transport
ALIX	Associated proteins ALG2-interacting protein X
ECM	Extracellular matrix
ISEV	International Society for Extracellular Vesicles
NTA	nanoparticle tracking analysis
DLS	Dynamic light scattering
hESC	Human embryonic stem cells
OEVs	Organoid-derived EVs
PEG-4MAL	Four-armed maleimide-capped polyethylene glycol
HSC	Hematopoietic stem cells
IBD	Inflammatory bowel disease
HSPCs	Hematopoietic stem/progenitor cells
TEM	Transmission electron microscopy cells
RD	Retinal degeneration
MSCs	Mesenchymal stem cells
M3DB	magnetic 3D bioassembly
GBM	Glioblastoma
CRA	Colorectal adenoma
CRC	Colorectal cancer
LAMA5	Laminin subunit alpha 5
SDCBP	Syntenin-1
TENA	Tenascin
TME	Tumor microenvironment
NCPs	NanoCulture plates
GLA	Grape-like aggregation
DDS	Dextran Sulfate Sodium
LPS	Lipopolysaccharide
NT-4	Neurotrophin-4
GDNF	Glial cell line-derived neurotrophic factor
DA	Dopaminergic
